# Reverse transcription recombinase polymerase amplification assay for rapid detection of canine associated rabies virus in Africa

**DOI:** 10.1371/journal.pone.0219292

**Published:** 2019-07-05

**Authors:** Jessica Coertse, Jacqueline Weyer, Louis H. Nel, Wanda Markotter

**Affiliations:** 1 Centre for Viral Zoonoses, Department of Medical Virology, Faculty of Health Sciences, University of Pretoria, Pretoria, South Africa; 2 The Centre for Emerging Zoonotic and Parasitic Diseases, National Institute for Communicable Diseases, a Division of the National Health Laboratory Services, Sandringham, South Africa; 3 Centre for Viral Zoonoses, Department of Biochemistry, Genetics and Microbiology, Faculty of Natural and Agricultural Sciences, University of Pretoria, Pretoria, South Africa; National Institute of Environmental Health Sciences (NIEHS), UNITED STATES

## Abstract

Rabies is a neglected disease mostly affecting the developing world. Accurate and reliable diagnostic and surveillance data forms the foundation for the formulation and monitoring of control strategies. Although various sensitive and specific tests are available for detection of rabies virus, implementation of these tests in low-resource settings are challenging and remains limited. In this study, we describe the developed of a reverse transcription recombinase polymerase amplification assay for the detection of rabies virus. The analytical sensitivity of this assay was determined to be 562 RNA copies and was performed in 20 minutes. The diagnostic sensitivity of the RT-RPA was 100% for detection of rabies virus in field samples. In conclusion, the RT-RPA assay allowed for very quick and sensitive detection of rabies virus and could be adapted for use in low-source settings.

## Introduction

Rabies is a fatal viral infection that causes progressive encephalitis in all mammals and is caused by 16 officially recognized viral species [1,2]. It is estimated that approximately 59 000 human deaths occur each year due to rabies and that over 99% of these are caused by rabies virus (RABV) transmitted by domestic dogs [3]. RABV is established worldwide in various hosts and can be divided into two phylogenic groups i.e. bat-related RABV and dog-related RABV. The latter group can be further divided into several major clades i.e. the cosmopolitan, Africa 2, Africa 3, Arctic-related, Asian and Indian subcontinent clades [4]. As rabies is 100% preventable the United Against Rabies collaboration; consisting of the World Health Organization (WHO), the World Organisation for Animal Health (OIE), the Food and Agriculture Organization of the United Nations (FAO) and the Global Alliance for Rabies Control (GARC); have compiled a global strategic plan in order to eliminate dog-mediated human rabies by 2030 [5] that emphasizes the need for accurate data. To assess the true burden of disease, routine and accurate diagnosis of rabies is required, this however, has been shown to be a major limitation in developing countries that are plagued by limited diagnostic capability that leads to poor surveillance, underreporting and misdiagnosis [6–10]. The current gold standard for rabies diagnosis is the fluorescent antibody test (FAT) recommended by the OIE and WHO [11,12]. This test relies on the visualization of RABV antigens in fresh or frozen brain material by fluorescent microscopy. Implementation of the FAT in resource-limited countries, where rabies diagnosis is most needed, is hindered by the high cost of acquiring and maintaining fluorescent microscopes, lack of trained technicians and difficulty in collection and preservation of fresh specimens [7–9]. More recently a streptavidin-biotin peroxidase based test, the direct rapid immunohistochemical test (dRIT) [13], has been recommended as an alternative to the FAT in resource-limited laboratories [11] with a few developing countries beginning to implement the dRIT as a primary test for rabies diagnosis [14].

Molecular techniques for rabies diagnosis have become more wide-spread and accepted [15] and has been shown to be more reliable for decomposed tissues than the FAT [16,17]. However, similar to the FAT, several factors have prevented its implementation in resource-limited settings including the high cost of equipment and reagents, electricity requirements, skilled technicians and the need to maintain the cold chain. As such, the implementation of diagnostic tests in the developing world will be reliant on resource availability and simplicity [7,15] with members of the United Against Rabies collaboration promoting a shift towards point-of-care (POC) diagnostic tools [5].

The use of lateral flow devices or rapid immunodiagnostic tests has been suggested as an alternative to laboratory testing. However, the evaluation of six different commercially available lateral flow devices indicated poor performance with regards to sensitivity and specificity [18]. Due to these limitations, further improvement and standardization of these tests were recommended before they could be considered as a potential POC test [18]. Recombinase polymerase amplification (RPA) is a molecular method that is initiated when recombinase binds to primers to from a nucleoprotein filament that forms a D-loop structure where homologous sequences are present in duplex DNA. The D-loop structure then initiates a strand exchange reaction. The recombinase can then disassemble from the nucleoprotein filament and is available for the next pair of primers. Recombinase disassembly then allows DNA polymerase to initiate synthesis from the free 3’ end of the primer. As polymerization continues, strands can then separate and form two duplexes after which the whole process is repeated [19,20]. RPA reactions are carried out at constant temperature (usually 37–42°C), the lyophilised reaction pellets have a shelf-life of several months and can be stored at room temperature with the option of different detection methods including lateral flow devices and portable incubation and detection instruments [20]. RPA has therefore been recommended as a potential POC test due to its affordability, simplicity, sensitivity, speed and minimal equipment requirements [21]. Its effectiveness as a POC test has been demonstrated for human immunodeficiency virus type 1 [22], and as a portable or field test for foot-and-mouth disease virus [23] and avian influenza A (H7N9) virus [24]. The potential of RT-RPA for use in enhanced surveillance for rabies has been shown in a previous proof-of-principle study [25] by evaluating a small panel (n = 33) of samples with a reported diagnostic sensitivity of 97% and limit of detection (LOD) of 1000 RNA copies.

This study aimed to develop an RT-RPA assay for the detection of lyssaviruses in animal samples from Africa. During assay design our main priority was focused on detection of dog-related RABV since it is responsible for the majority of human rabies cases. This assay was shown to be quick and simple with detection of rabies virus within 20 minutes, sensitivity of 562 RNA copies and specificity of 100%. Furthermore, the RT-RPA assay shows promise for further development as a pan-lyssavirus assay for potential use in low-resource settings.

## Material and methods

### Ethics statement and samples

This research was conducted with the approval of the University of Pretoria Animal Ethics Committee (Project number: H005-16). A panel of brain material (n = 109) collected from naturally infected animals [8,9,26–29] that tested positive with the FAT [30] and the direct, rapid immunohistochemical test (DRIT) [8] and four negative samples were included for diagnostic evaluation of the RT-RPA. RNA was extracted from all samples (from approximately 50 mg tissue) using Trizol reagent (Invitrogen) according to the manufacturer’s instructions and eluted in 50 μl nuclease free water (Ambion). To determine cross-reactivity of the assay, RNA from 18 virus cell culture supernatants were also included.

### qRT-PCR

The One Step PrimeScript RT-PCR kit (Takara Bio Inc) was used for all qRT-PCR reactions on a QuantStudio 5 real-time PCR system (Thermo Fisher Scientific) as described previously [31,32] with modifications. Briefly, 1 μl extracted RNA was amplified in a final volume of 10 μl containing 1x One step RT-PCR Buffer III, 1U TaKaRa Ex Taq HS, 0.2 μl PrimeScipt RT enzyme mix II, 0.8 μM of each primer (541lys: 5’-CACMGSNAAYTAYAARACNAA-3’ and 550B: 5’-GTRCTCCARTTAGCRCACAT-3’) and 0.4 μM probe 620lyssaC (5’: 6-carboxyfluorescein (FAM)–CAYCAYACHYTVATGACHACHCAYAA–non-fluorescent quencher (QSY) 3’). First-strand synthesis was achieved by incubation at 42°C for 30 minutes and subsequent denaturation at 95°C for 10 seconds. Reactions were cycled 45 times at 95°C for 5 seconds, 42°C for 5 seconds and 72°C for 5 seconds. Viral RNA copy numbers were estimated using external standard curves as described previously [31,32] using the QuantStudio Design and Analysis Software version 1.4 (Thermo Fisher Scientific).

### Generation of standard RNA

The complete nucleoprotein gene of challenge virus standard (CVS-11) was amplified using primers Lys001 (5’-ACGCTTAACGAMAAA-3’) and 304 (5’-TTGACAAAATCTTCTCAT-3’) as described previously [33]. Amplification products were purified using the Zymoclean Gel DNA Recovery Kit (Zymo Research) followed by cloning using the pGEM-T Easy vector system (Promega) according to the manufacturer’s instructions. Recombinant clones were characterized by sequencing using the BigDye Terminator V3.1 cycle sequencing kit (Thermo Fisher Scientific) and an ABI3500xL genetic analyser (Applied Biosystems) to determine orientation. A single recombinant clone containing the insert in the correct orientation with regard to the SP6 promoter was selected, and the insert was *in vitro* transcribed using the MegaScript kit (Ambion) according to the manufacturer’s instructions. *In vitro* transcribed RNA was purified using the RNA Clean and Concentrator-25 kit (Zymo Research) and quantified using the Qubit 3.0 fluorometer (Invitrogen). Contamination with plasmid DNA was ruled out with no-RT controls.

### RT-RPA

#### Primer and probe design

The ClustalW subroutine of BioEdit Sequence Alignment Editor version 7 [34] was used to create a multiple alignment of representative sequences for RABV available on GenBank (https://www.ncbi.nlm.nih.gov/) (S1 Table). Regions of homology were identified, and primers and probes were designed targeting the nucleoprotein gene.

#### RT-RPA reaction conditions

All RT-RPA reactions were performed using the TwistAmp exo RT kit (TwistDx) and optimized regarding magnesium acetate concentration (MgOAc), time of shaking, probe concentration, primer selection and concentration. The optimized master mix (46.16 μl) contained 600 nM of each primer (RPA_RV_N497F and RPA_RV_N681R, Table 1), 200 nM exo-probe (Table 1), 40U RiboLock RNase Inhibitor (Thermo Scientific) and 29.5 μl rehydration buffer that was added to the lyophilized reaction pellet followed by the addition of 1 μl RNA. The reaction was initiated by the addition of 2.86 μl magnesium acetate (280 mM). The reactions were incubated for a total of 20 minutes at 42°C in an ESEQuant Tube Scanner (Qiagen) with brief vortexing after 6 minutes. Fluorescent signal (FAM channel) was measured at 20 second intervals and analyzed using the Tube Scanner Studio software (Qiagen) with regards to threshold validation (fluorescence increases three standard deviations above the background during the first minute of the reaction) and slope validation (set at 15 mV/min) that was verified with calculation of the second derivative.

**Table 1:** Details of probe and primers used in the RT-RPA assay.

Primer or probe	Sequence (5’-3’)	Position on genome^g^
RPA_RV_N461F^a^	CAGGACAAAACACCGGCAACTATAAGACAAAC	461
**RPA_RV_N497F**^**b**^	CAGATAGGATAGAGCAGATTTTCGAGACAGC	497
RPA_RV_N528F	CCCCTTTTGTTAAAATCGTGGAACACCATAC	528
RPA_RV_N557F	ACAAACATYGCRGATAGRATAGAGCAGATTTTY	488
RPA_RV_N645R	CTGATTGCTGAATATCTCTGCTCAATCCGG	645
**RPA_RV_N681R**	GAGCAGTCTTCATAAGCAGTGACAACTGTG	681
RPA_RV_N692R	GYTCAATCCGGGAGAAAWACATGTCRTTTCC	622
**RPA_RV_N562exoprobe**	ATGACAACTCAYAARATGTGYGCYAATTGGAGYAC(FAMdTc^c^/THFd^d^/BHQ-1dTe^e^)ACCRAAYTT(C3Sp^f^)	562

^a^ R: reverse primer

^b^ F: forward primer

^c^ FAMdTC: thymidine nucleotide carrying 6-carboxyfluorescein

^d^ THFd: tetrahydrofuran residue

^e^ BHQ-1dTe: thymidine nucleotide carrying black hole quencher 1

^f^ C3Sp: C3 spacer to block elongation

^g^ Nucleotide positions are numbered according to rabies virus, CVS-11 (GenBank accession number GQ918139)

#### RT-RPA assay characteristics

The analytical sensitivity of the RT-RPA assay was determined by testing serially diluted i*n vitro* transcribed rabies virus (CVS-11) RNA (200, 300, 500, 10^3^−10^8^ copies). Probit regression analysis was performed on four replicates of the various dilutions using MedCalc Statistical software version 18.10.2 (MedCalc Software bvba). Diagnostic sensitivity and specificity were evaluated by testing rabies positive and negative brain material from Africa (Table 2) with RT-RPA and compared with qRT-PCR. Additionally, RNA representing different rabies-related viruses and bat-related RABV lineages (Table 3) were also tested with both methods to determine cross-reactivity.

**Table 2:** Detection of different rabies positive and negative field samples using RT-RPA and qRT-PCR.

Laboratory number	Country	Host	RT-RPA		qRT-PCR			
			Result	Time	Result	Copy nr^h^	Cp	Time^g^
99/14^a^	Lesotho	Canine	Pos	5	Pos	1,90E7	14,8	46,8
60/14^a^	Lesotho	Bovine	Pos	5	Pos	2,27E6	18,2	50,5
45a/14^a^	Lesotho	Bovine	Pos	4	Pos	2,92E7	14,2	46,0
45b/14^a^	Lesotho	Bovine	Pos	3,7	Pos	6,69E7	12,9	44,6
08/14^a^	Lesotho	Bovine	Pos	3,7	Pos	9,13E7	12,4	44,1
195/14^a^	Lesotho	Bovine	Pos	3,7	Pos	6,63E7	12,9	44,6
190/12^a^	Lesotho	Caprine	Pos	4	Pos	3,62E7	13,8	45,7
21/15^a^	Lesotho	Bovine	Pos	15,3	Pos	4,00E4	24,6	57,5
24/15^a^	Lesotho	Canine	Pos	5,3	Pos	4,86E5	20,6	53,2
17/15^a^	Lesotho	Canine	Pos	5,7	Pos	5,06E5	20,6	53,1
22/16^a^	Lesotho	Bovine	Pos	5,3	Pos	8,83E5	19,7	52,1
298/93^b^	Mozambique	Canine	Pos	5	Pos	4,00E5	20,9	53,5
186/99^b^	Mozambique	Canine	Pos	5,3	Pos	3,90E5	21,9	54,6
572/99^b^	Mozambique	Canine	Pos	4,7	Pos	8,50E5	19,8	52,2
633/00^b^	Mozambique	Canine	Pos	5,3	Pos	9,04E5	19,7	52,1
315/04^b^	Mozambique	Canine	Pos	5,3	Pos	3,19E5	21,3	53,9
232/05^b^	Mozambique	Canine	Pos	8,7	Pos	1,24E4	26,4	59,5
558/05^b^	Mozambique	Canine	Pos	5,3	Pos	6,13E5	20,3	52,8
659/05^b^	Mozambique	Canine	Pos	4,3	Pos	9,38E5	19,6	52,0
687/05^b^	Mozambique	Canine	Pos	4	Pos	5,59E5	20,4	52,9
131/12^b^	Mozambique	Feline	Pos	5,3	Pos	1,06E6	19,4	51,8
482/12^b^	Mozambique	Canine	Pos	4,7	Pos	1,36E6	19,0	51,4
1018/12^b^	Mozambique	Bovine	Pos	4,3	Pos	1,26E6	19,1	51,5
233/13^b^	Mozambique	Canine	Pos	5	Pos	1,35E6	19,0	51,4
191K09^c^	Namibia	Kudu	Pos	3,7	Pos	4,03E7	13,7	45,5
212K09^c^	Namibia	Kudu	Pos	4	Pos	2,45E7	14,4	46,4
234K09^c^	Namibia	Kudu	Pos	5,3	Pos	1,42E7	15,3	47,3
240K09^c^	Namibia	Kudu	Pos	1	Pos	8,08E5	19,8	52,3
151J09^c^	Namibia	Jackal	Pos	5	Pos	3,47E7	13,9	45,8
179J09^c^	Namibia	Jackal	Pos	4	Pos	8,58E7	12,5	44,2
192J09^c^	Namibia	Jackal	Pos	3	Pos	1,78E8	11,3	42,9
193J09^c^	Namibia	Jackal	Pos	4	Pos	3,64E6	17,5	49,7
197J09^c^	Namibia	Jackal	Pos	8	Pos	4,34E5	20,8	53,4
204J09^c^	Namibia	Jackal	Pos	5,7	Pos	1,39E6	19,0	51,3
236J09^c^	Namibia	Jackal	Pos	4	Pos	1,42E7	15,3	47,3
NIG49^d^	Nigeria	Canine	Pos	18,3	Pos	7,90E8	8,9	40,3
NIG50^d^	Nigeria	Canine	Pos	4,3	Pos	4,92E8	9,7	41,2
NIG65^d^	Nigeria	Canine	Pos	2	Pos	2,66E8	10,7	42,2
NIG95^d^	Nigeria	Canine	Pos	1	Pos	1,11E8	12,0	43,7
NIG140^d^	Nigeria	Canine	Pos	3,3	Pos	1,77E8	11,3	42,9
NIG142^d^	Nigeria	Canine	Pos	3,3	Pos	6,52E8	9,3	40,7
NIG176^d^	Nigeria	Canine	Pos	8	Pos	1,62E8	11,5	43,1
NIG185^d^	Nigeria	Canine	Pos	3,7	Pos	5,97E8	9,4	40,8
NIG249^d^	Nigeria	Canine	Pos	9	Pos	2,53E8	10,7	42,3
NIG251^d^	Nigeria	Canine	Pos	8,3	Pos	5,83E8	9,4	40,9
71/14^e^	Zimbabwe	Canine	Pos	4,7	Pos	1,21E7	15,6	47,6
168/14^e^	Zimbabwe	Feline	Pos	15,3	Pos	2,67E3	28,9	62,2
307/15^e^	Zimbabwe	Canine	Pos	13	Pos	6,28E6	16,6	48,7
406/15^e^	Zimbabwe	Canine	Pos	4,7	Pos	4,27E7	13,6	45,4
424/15^e^	Zimbabwe	Canine	Pos	4	Pos	2,23E7	14,6	46,5
427/15^e^	Zimbabwe	Canine	Pos	14,7	Pos	9,51E7	12,3	44,0
464/15^e^	Zimbabwe	Canine	Pos	4	Pos	3,70E7	13,8	45,6
515/15^e^	Zimbabwe	Canine	Pos	5	Pos	1,18E7	15,6	47,6
245/16^e^	Zimbabwe	Bovine	Pos	7,7	Pos	4,59E5	20,7	53,3
12/526^f^	South Africa	Caprine	Pos	3,7	Pos	6,21E7	13,0	44,7
12/621^f^	South Africa	Jackal	Pos	4,7	Pos	1,63E6	18,7	51,1
12/616^f^	South Africa	Jackal	Pos	4,7	Pos	2,38E6	18,1	50,4
12/730^f^	South Africa	Canine	Pos	3,3	Pos	8,42E7	12,5	44,2
12/849^f^	South Africa	Bovine	Pos	17,3	Pos	2,08E3	29,3	62,6
15/17^f^	South Africa	Canine	Pos	4,3	Pos	1,59E7	15,1	47,1
15/142^f^	South Africa	Mongoose	Pos	4,3	Pos	1,26E7	15,5	47,5
15/170^f^	South Africa	Mongoose	Pos	1	Pos	1,12E8	12,0	43,7
15/173^f^	South Africa	Mongoose	Pos	8	Pos	2,24E8	10,9	42,5
15/181^f^	South Africa	Duiker	Pos	5	Pos	4,87E6	17,0	49,2
15/182^f^	South Africa	Lynx	Pos	5	Pos	2,67E7	14,3	46,2
15/295^f^	South Africa	Bovine	Pos	3,7	Pos	5,11E7	13,3	45,1
15/479^f^	South Africa	Bovine	Pos	5	Pos	1,17E6	19,2	51,6
15/495^f^	South Africa	Canine	Pos	3,7	Pos	3,97E7	13,7	45,5
15/305^f^	South Africa	Bovine	Pos	3,7	Pos	1,55E7	15,2	47,1
15/538^f^	South Africa	Bovine	Pos	4,3	Pos	3,61E7	13,8	45,7
15/543^f^	South Africa	Canine	Pos	5,7	Pos	5,06E5	20,6	53,1
15/553^f^	South Africa	Otter	Pos	5	Pos	1,33E7	15,4	47,4
15/563^f^	South Africa	Canine	Pos	3,7	Pos	1,50E6	18,9	51,2
15/595^f^	South Africa	Jackal	Pos	3,7	Pos	5,02E6	13,3	45,1
15/634^f^	South Africa	Mongoose	Pos	4	Pos	3,36E7	13,9	45,8
15/636^f^	South Africa	Bovine	Pos	4	Pos	4,20E6	17,2	49,4
15/643^f^	South Africa	Canine	Pos	4,7	Pos	4,19E6	17,2	49,4
15/644^f^	South Africa	Bovine	Pos	4	Pos	8,50E8	12,5	44,2
16/010^f^	South Africa	Jackal	Pos	4,7	Pos	3,76E6	17,4	49,6
16/051^f^	South Africa	Canine	Pos	8	Pos	3,09E5	21,4	53,9
16/069^f^	South Africa	Jackal	Pos	5,7	Pos	2,03E6	18,4	50,7
16/094^f^	South Africa	Mongoose	Pos	4,3	Pos	3,29E6	17,6	49,8
16/102^f^	South Africa	Jackal	Pos	4,7	Pos	8,27E6	16,2	48,2
17/298^f^	South Africa	Mongoose	Pos	3,3	Pos	1,84E8	11,3	42,9
17/330^f^	South Africa	Mongoose	Pos	4,7	Pos	4,44E6	17,1	49,3
17/336^f^	South Africa	Mongoose	Pos	3,3	Pos	6,99E8	9,1	40,5
15/304^d^	South Africa	Jackal	Pos	4	Pos	8,48E5	19,8	52,2
15/467^d^	South Africa	Canine	Pos	14,3	Pos	6,69E6	16,5	48,6
15/474^d^	South Africa	Canine	Pos	14,3	Pos	7,23E6	16,4	48,5
15/519^d^	South Africa	Bovine	Pos	14	Pos	9,19E6	16,6	48,7
16/239^d^	South Africa	Bovine	Pos	4,7	Pos	4,50E7	13,5	45,3
16/247^d^	South Africa	Canine	Pos	3,7	Pos	8,37E7	12,5	44,2
16/256^d^	South Africa	Caprine	Pos	3,3	Pos	1,30E8	11,8	43,5
16/260^d^	South Africa	Ovine	Pos	4	Pos	9,62E7	12,3	44,0
16/286^d^	South Africa	Bovine	Pos	4	Pos	4,26E7	13,6	45,4
16/318^d^	South Africa	Canine	Pos	5,3	Pos	2,49E7	14,4	46,3
16/343^d^	South Africa	Bovine	Pos	4,3	Pos	4,46E7	13,5	45,3
14/406^d^	South Africa	Caprine	Pos	5	Pos	1,37E8	11,7	43,4
14/424^d^	South Africa	Canine	Pos	4	Pos	2,80E8	10,6	42,1
15/130^d^	South Africa	Bovine	Pos	4,3	Pos	1,84E7	14,9	46,9
15/205^d^	South Africa	Canine	Pos	5	Pos	1,51E7	15,2	47,2
13/339^f^	South Africa	Canine	Pos	1	Pos	1,88E8	11,2	42,8
13/310^f^	South Africa	Canine	Pos	1	Pos	3,87E5	21,0	53,6
13/107^f^	South Africa	Canine	Pos	3	Pos	5,25E8	9,6	41,0
13/522^f^	South Africa	Canine	Pos	1	Pos	9,03E7	12,4	44,1
13/256^f^	South Africa	Canine	Pos	1,3	Pos	4,18E8	10,0	41,4
13/355^f^	South Africa	Canine	Pos	3,7	Pos	2,76E8	10,6	42,2
13/104^f^	South Africa	Canine	Pos	1	Pos	3,14E8	10,4	41,9
13/79^f^	South Africa	Canine	Pos	1	Pos	2,33E8	10,9	42,4
RK002	South Africa	Jackal	Neg		Neg			
RK010	South Africa	Fox	Neg		Neg			
RK018	South Africa	Civet	Neg		Neg			
RK023	South Africa	Canine	Neg		Neg			

^a^ Obtained from the Central Veterinary Laboratory, Maseru, Lesotho; samples positive with the FAT [30] and DRIT [8]

^b^ Obtained from the Central Veterinary Laboratory, Maputo, Mozambique; samples positive with the FAT [30] and DRIT [8]

^c^ Obtained from the Central Veterinary Laboratory, Windhoek, Namibia; samples positive with the FAT [30] and DRIT [8]

^d^ Obtained from the Agricultural Research Council-Onderstepoort Veterinary Research, Gauteng Province, South Africa; samples positive with the FAT [30] and DRIT [8]

^e^ Obtained from the Central Veterinary Laboratory, Harare, Zimbabwe; samples positive with the FAT [30] and DRIT [8]

^f^ Obtained from Allerton Provincial Veterinary Laboratory, KwaZulu-Natal Province, South Africa; samples positive with the FAT [30] and DRIT [8]

^g^ For better comparability between assays, qRT-PCR crossing point values were converted into estimated detection time

^h^ RNA copies/μl eluate

**Table 3:** Detection of RNA from representative lyssavirus species using RT-RPA and qRT-PCR.

		RT-RPA		qRT-PCR			
Lyssavirus species	Country	Result	Time	Result	Copy nr	Cp	Time^a^
ARAV	Kyrgyzstan	Pos	8	Pos	2.7E8	10.7	42.2
DUVV	South Africa	Neg		Pos	9,0E7	12.4	44.1
EBLV-1	Denmark	Neg		Pos	4.2E6	17.2	49.4
EBLV-2	United Kingdom	Pos	18.7	Pos	4.2E7	13.6	45.4
IKOV	Tanzania	Neg		Pos	2.7E6	17.9	50.1
IRKV	Russia	Pos	16.7	Pos	2.4E8	10.9	42.4
KHUV	Tajikistan	Pos	18.7	Pos	6.1E8	9.4	40.8
LBV (lineage A)	Unknown	Pos	15.3	Pos	2.5E8	10.8	42.3
LBV (lineage C)	South Africa	Pos	19	Pos	1.2E7	15.6	47.6
LBV (lineage D)	Kenya	Pos	16.7	Pos	4.3E5	20.8	53.4
MOKV	South Africa	Pos	5.7	Pos	1.7E6	18.7	51
RABV (free-tailed bat strain)	Americas	Pos	3,7	Pos	9,2E6	15.2	47.2
RABV (silver-haired bat strain)	Americas	Pos	15.7	Pos	1.1E7	15.6	47.6
RABV (Vampire strain)	Americas	Pos	15	Pos	5.8E7	12.2	43.9
RABV (Eastern big brown bat strain)	Americas	Pos	15.3	Pos	1.2E8	11.9	43.5
RABV (Western big brown bat strain)	Americas	Pos	17	Pos	5.5E7	13.2	45
SHIBV	Kenya	Neg		Pos	2.1E5	21.4	54
WCBV	Russia	Pos	15.3	Pos	2.5E8	10.8	48.7

^a^ For better comparability between assays, qRT-PCR crossing point values were converted into estimated detection time

## Results

### RT-RPA Primer evaluation and assay optimization

For the development of a rabies RT-RPA assay, seven primers (four forward and three reverse) and a degenerate RPA exo probe (Table 1) were designed and evaluated using conditions recommended by the manufacturer.

To identify the optimal primer pair, a total of seven primer pairs were evaluated using *in vitro* transcribed CVS RNA (10^8^ copies/μl) with final RT-RPA amplicon lengths ranging from 117–220 bp. All primer sets were able to amplify rabies virus (CVS-11) RNA, albeit with different efficiencies and were detected within 6 minutes. Primer set RPA_RV_N497F and RPA_RV_N681R performed the best with detection after 2.7 minutes and was subsequently used for further evaluation of the assay.

### Analytical sensitivity of the RT-RPA assay

**Fig 1:**
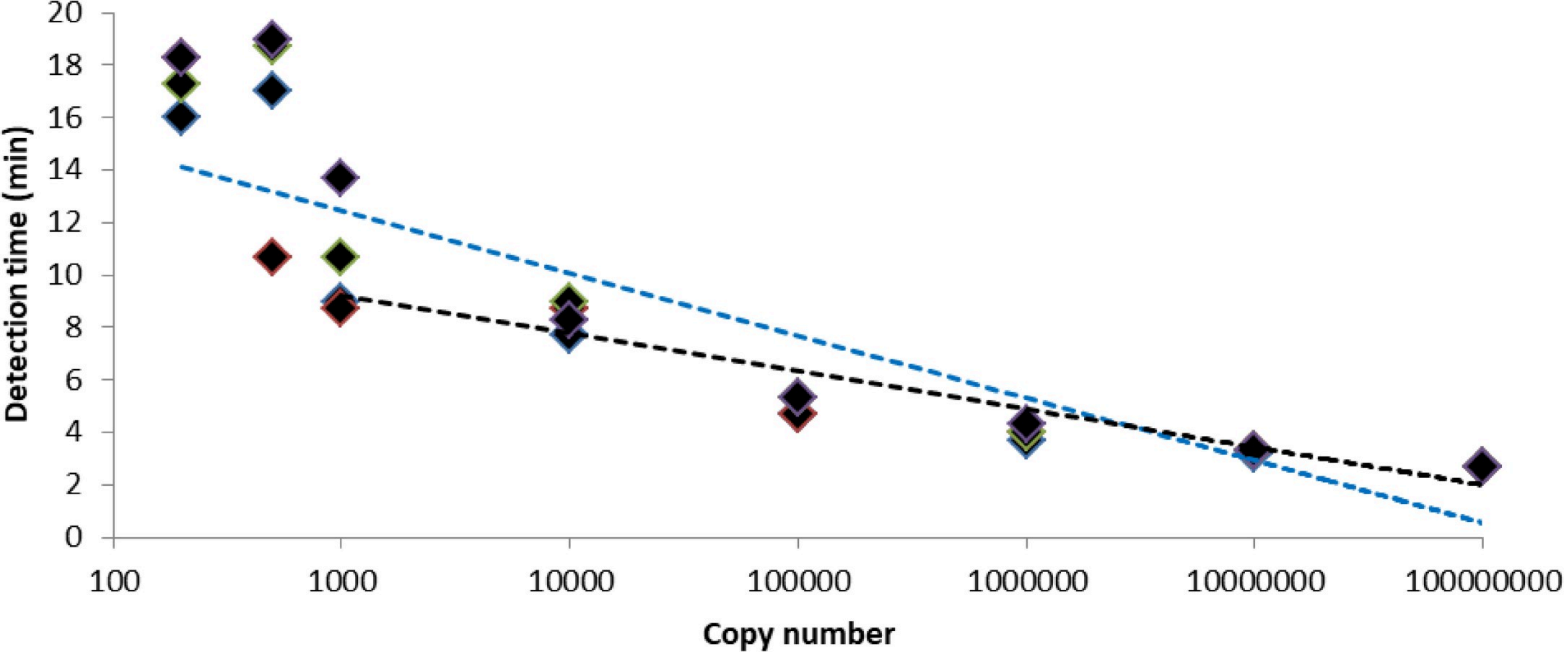
RT-RPA performance. Detection times of four replicates of serially diluted *in vitro* transcribed rabies virus (CVS-11) RNA. Correlation between detection time and copy number is indicated by semi-logarithmic regression lines of all dilutions evaluated i.e. 200−10^8^ copies (blue dotted line, R^2^ = 0.82) and where the two lowest dilutions were excluded i.e. 10^3^−10^8^ copies (black dotted line, R^2^ = 0.93).

Serially diluted *in vitro* transcribed rabies virus (CVS-11) RNA was used to determine the analytical sensitivity of the RT-RPA assay using optimized conditions. The calculated LOD using probit regression analysis at 95% probability was 562 RNA copies (95% confidence interval: 331–933 RNA copies). Good correlation was seen between copy number and time to positive (R^2^ = 0.93) when 10^3^−10^8^ copies were considered, however, this correlation decreased when lower copy numbers (<10^3^ copies) were included (Fig 1).

### Diagnostic sensitivity of the RT-RPA assay

**Fig 2:**
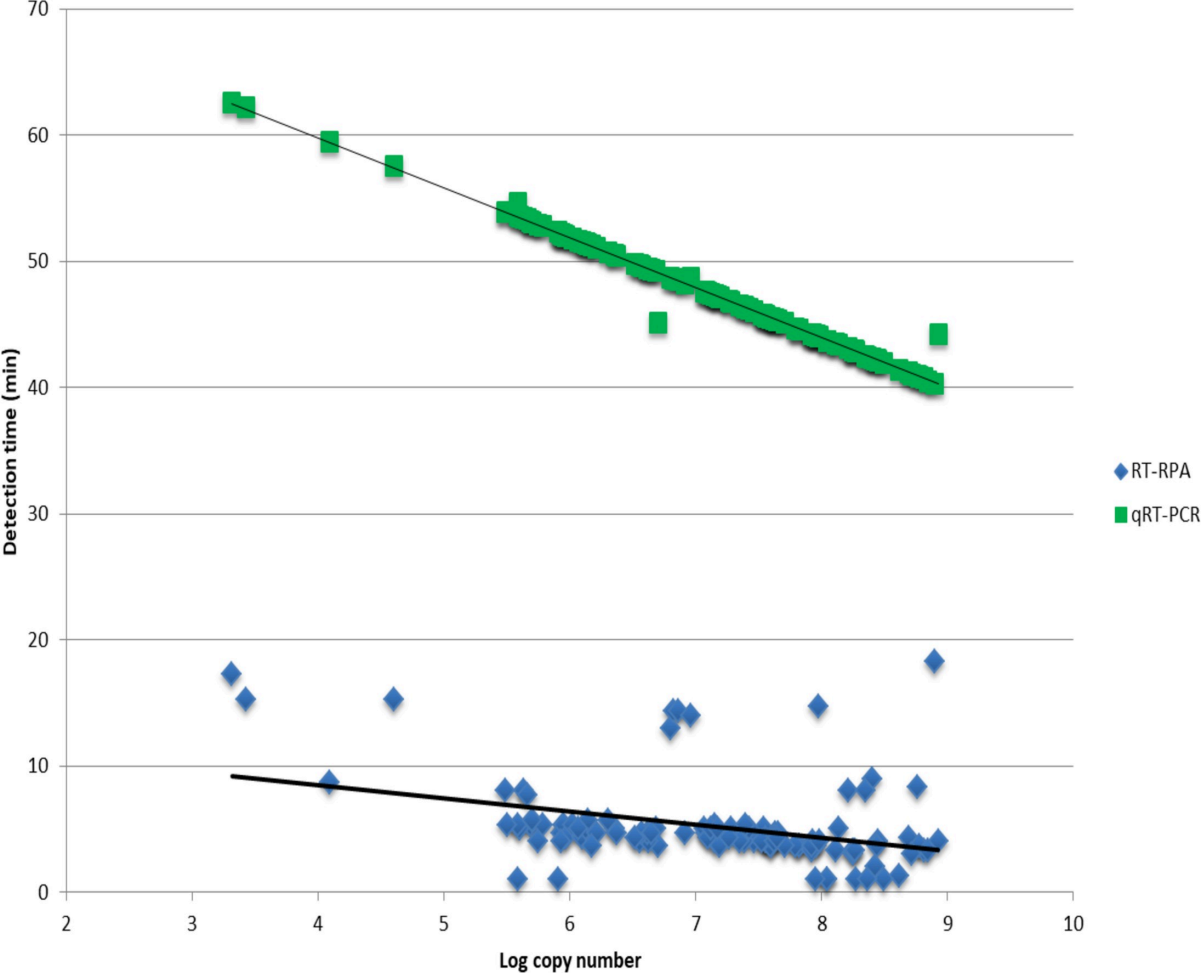
Correlation between estimated copy number and detection time of field samples tested with RT-RPA and qRT-PCR. Linear regression analysis of RT-RPA and qRT-PCR detection times and estimated copy numbers for 109 rabies positive brain material.

The RT-RPA assay was evaluated by testing a cohort of rabies positive and negative samples and compared to qRT-PCR (Table 2). All samples were detected with qRT-PCR and the RT-RPA resulting in a diagnostic sensitivity of 100%. The average detection time of the RT-RPA was 5.3 minutes compared to 47.6 minutes for qRT-PCR. Good correlation was observed for qRT-PCR copy number and detection time (R^2^ = 0.99), however, linear regression analysis demonstrated poor correlation between estimated copy number and RT-RPA detection time (R^2^ = 0.13, Fig 2). This poor correlation indicates that RT-RPA detection time should not be applied to quantitative analysis.

To determine if the RT-RPA assay could be used as a pan-lyssavirus detection assay, representatives of 11 lyssavirus species and representatives of bat-related RABV lineages were tested (Table 3). The RT-RPA showed cross detection of 7 lyssavirus species, i.e., ARAV, EBLV-2, IRKV, KHUV, LBV, MOKV, WCBV and could detect 5 bat-related RABV lineages from the Americas. However, detection with the RT-RPA occurred much later than expected for the majority of samples (compared to the estimated copy number determined with qRT-PCR).

**Fig 3:**
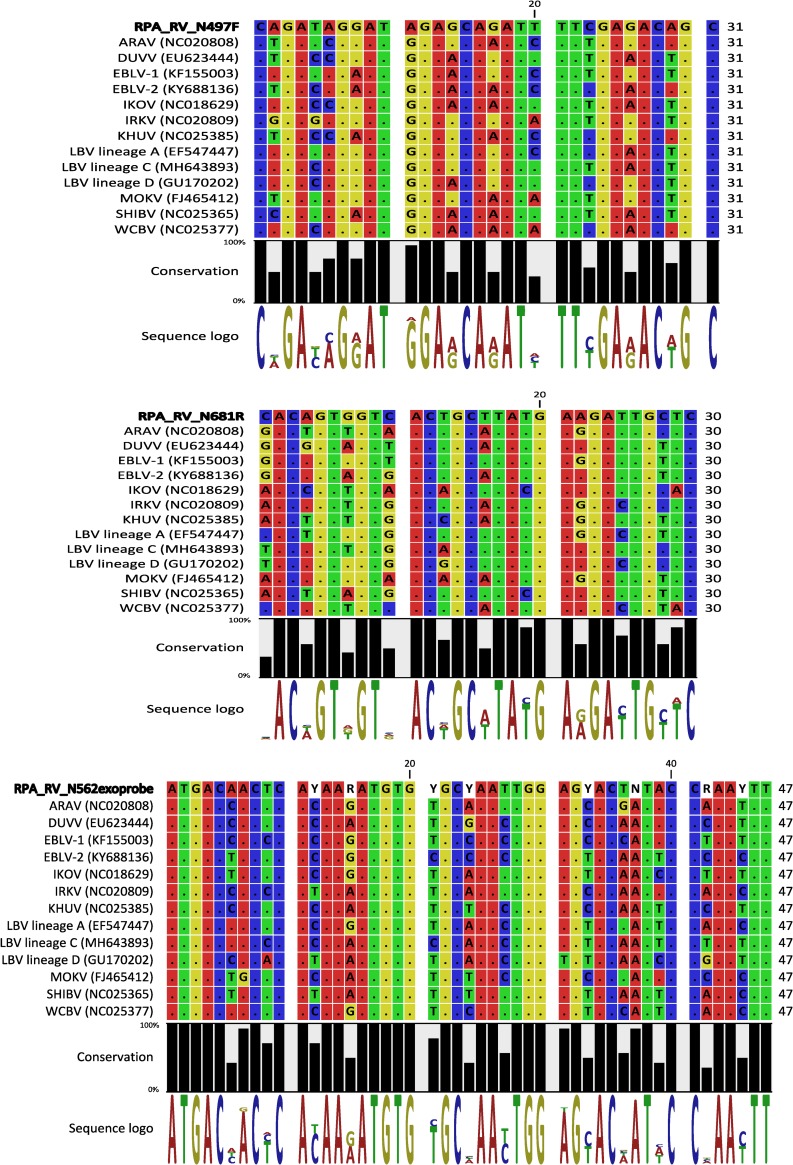
Binding regions of the RT-RPA primers and probe to rabies-related viruses. Dots represent identity to the sequence in the first row i.e. RT-RPA forward primer (RPA_RV_N497F), reverse complement of the reverse primer (RPA_RV_N681R) and the probe (RPA_RV_N562exoprobe). The conservation percentage and the sequence logo created using CLC Genomic Workbench software version 6 (Qiagen) is displayed beneath the alignment. The conservation graph shows the conservation percentage of all sequence positions, the height of the bars shows how conserved that particular position is in the alignment. The sequence logo displays the information content of all positions in the alignment as nucleotides stacked on top of each other. The height of the individual letters represents the sequence information content in that particular position of the alignment.

Evaluation of the probe and primer binding regions indicated 4–8 mismatches with the primers and 2–7 mismatches with the probe (Fig 3). In general, mismatches up to 14% were tolerated by the assay and detection failure was only noted when this value was exceeded (S2 Table).

## Discussion

The lack of inexpensive, rapid and simple diagnostic tests has been cited as a major constraint for assessment of the true burden of rabies [7]. Molecular-based methods have received increasing attention in recent years with several assays for broad-spectrum or targeted detection of lyssaviruses (reviewed in [35]) and have been indicated to be more sensitive than the gold standard, FAT [36]. Molecular methods, such as RT-PCR and real-time RT-PCR, although sensitive and specific, rely on constant power supply, expensive equipment, skilled personnel and sophisticated laboratories. To overcome some of these obstacles isothermal molecular techniques have been developed such as loop-mediated isothermal amplification (LAMP) and nucleic acid sequence-based amplification (NASBA). Several RT-LAMP assays have been used for rabies virus detection [37–40] and has been shown to be a quick (>1 hour) and sensitive (approximately 1000 RNA copies) method, however, this assay is limited by the considerable sequence variation between and within lyssavirus species that can make designing broadly reactive primer sets difficult [15,40]. In contrast, primer design for NASBA is relatively simple, but this assay requires longer running times (2-3h) and is more expensive than other molecular methods [41].

RPA has been identified as a promising tool for the quick, cost-effective identification of pathogens with several assays being developed that can detect a wide variety of RNA and DNA pathogens (reviewed in [42]) including rabies virus [25]. In this study, we developed and evaluated an RPA assay for the detection of rabies, with a specific focus on dog-related RABV in Africa. The length of RPA primers exceeds that of standard PCR primers and could, therefore, be problematic for variable viruses. Additionally, the performance of various primer sets in RPA assays cannot be determined *a priori* and should be experimentally evaluated, the influence of the number and distribution of mismatches with target sequences is also not well understood [43]. Thus, several primer sets based on the conserved nucleoprotein gene were designed, according to the kit manufacturer’s website (http://www.twistdx.co.uk), including a degenerate primer set to compensate for variability. The combination of non-degenerate primers RPA_RV_N497F and RPA_RV_N681R was shown to be the most effective for amplification and yielded the highest sensitivity of 562 RNA copies as determined with probit regression analysis. This assay shows improved sensitivity compared to previously published isothermal methods, i.e., RT-LAMP [40] and RT-RPA [25] assays (1000 RNA copies). The diagnostic sensitivity of this assay was 100% for a sample cohort collected across Africa. Samples on average were detected in <6 minutes with estimated RNA copy numbers ranging from 2080 to >10^8^. The sensitivity of the assay (LOD of 562 RNA copies) would therefore be adequate for use as a rabies diagnostic tool using brain material. Linear regression analysis demonstrated poor correlation between estimated copy number and RT-RPA detection time. RT-RPA has been reported to produce non-linear curves that are unsuitable for quantification [23,44,45]. Several explanations have been proposed for this observation including the use of a chemical start (addition of magnesium acetate) rather than a thermal start (as with PCR assays) for the reaction; and since no thermal cycling is employed in RPA, synchronization is absent and annealing will occur continuously resulting in quantitative variation when using real-time fluorescent probes [20]. To determine the applicability of the current assay as a pan-lyssavirus surveillance tool, several lyssavirus species were tested. All bat-related RABV lineages (from the Americas) were detected, but only 64% of the rabies-related lyssaviruses were detected with the RT-RPA. Detection times of the RT-RPA occurred much later than expected for almost all rabies-related viruses (except MOKV). This indicates that the assay in its current form has low replication efficiency and would not be sensitive enough for use as a surveillance tool for rabies-related lyssaviruses or bat-related RABV. A previous study reported that up to 8% mismatches across primer pairs were tolerable [22] and comparable results (11% mismatches) were obtained when evaluating 87 primers [43]. Although no direct correlation between the amount and location of mismatches and RPA amplification could be determined, it was shown that mismatches at the 3’ end of both primers usually resulted in reduced efficiency [43]. Evaluating the mismatches between the RT-RPA primers and probe indicated mismatches of 13–26% and 2–15% respectively to rabies-related viruses. However, similar to Daher et al. [43], no correlation was observed between the number and location of mismatches and detection failure/success. Nevertheless, these results indicate the moderate tolerability of RPA to polymorphisms. This feature could lend itself towards the development of pan-lyssavirus or region-specific lyssavirus assays by modification of the primers described or inclusion of multiple primer sets in the reaction.

In conclusion, we developed an RT-RPA assay that was shown to be sensitive and specific for the detection of dog-related RABV in Africa. The assay demonstrated 100% diagnostic sensitivity compared to an established qRT-PCR. Although the sample cohort tested do not cover the full genetic diversity of RABV, the simplified approach and reduced turnaround time (approximately 9 times faster than qRT-PCR) of this assay suggests that it should be considered as a supplementary tool, where basic laboratory infrastructure is available, for enhanced surveillance efforts that could contribute towards rabies control efforts in a timeous manner. The TwistAmp exo RT kit (TwistDx) is no longer available; however, the assay described can be reproduced by adding a reverse transcriptase enzyme (such as Murine Leukemia virus reverse transcriptase) to the TwistAmp exo kit. This assay also shows promise as a valuable tool and possibly portable test for rabies diagnosis in resource-limited settings pending further development and evaluation.

## Supporting information

S1 Table Details of rabies virus sequences used for primer and probe design for the RT-RPA assay. (DOCX)

S2 Table Evaluation of the mismatches between the RT-RPA primer and probe set binding regions to rabies-related lyssaviruses. (DOCX)
