# Supplementary material for: Reverse transcription recombinase polymerase amplification assay for rapid detection of canine associated rabies virus in Africa
Source: PLoS One. 2019 Jul 5;14(7):e0219292. doi: 10.1371/journal.pone.0219292 (PMC6611627; doi:10.1371/journal.pone.0219292)
Supplement: S1 Table — (DOCX) [file pone.0219292.s001.docx]

**S1 Table. Details of rabies virus sequences used for primer and probe design for the RT-RPA assay**

| Genbank accession number | Year | Country | Host | Clade |
| --- | --- | --- | --- | --- |
| EU853612 | 2007 | Mali | Dog | Africa 2 |
| EU853615 | 2007 | Ivory Coast | Dog | Africa 2 |
| EU478515 | 2007 | Burkina Faso | Dog | Africa 2 |
| EU853637 | 2003 | Senegal | Human | Africa 2 |
| EU853651 | 2004 | Central African Republic | Dog | Africa 2 |
| EU853591 | 2008 | Gambia | Dog | Africa 2 |
| EU478515 | 2007 | Burkina Faso | Dog | Africa 2 |
| EU853637 | 2003 | Senegal | Human | Africa 2 |
| EU853612 | 2007 | Mali | Dog | Africa 2 |
| EU853591 | 2008 | Gambia | Dog | Africa 2 |
| EU718735 | 2005 | Chad | NA | Africa 2 |
| U22485 | 1985 | Benin | Cat | Africa 2 |
| U22486 | 1986 | Burkina Faso | Dog | Africa 2 |
| U22489 | 1986 | Mauritania | Camel | Africa 2 |
| U22635 | 1988 | Cameroon | Cat | Africa 2 |
| U22641 | 1990 | Guinea | Dog | Africa 2 |
| U22644 | 1992 | Chad | Dog | Africa 2 |
| U22652 | 1992 | Ivory Coast | Dog | Africa 2 |
| FJ392369 | 2000 | South Africa | Slender mongoose | Africa 3 |
| FJ392370 | 2000 | South Africa | Cattle | Africa 3 |
| FJ392376 | 2001 | South Africa | Sheep | Africa 3 |
| FJ392367 | 2001 | South Africa | Cat | Africa 3 |
| FJ392368 | 2001 | South Africa | Suricate | Africa 3 |
| FJ392382 | 2002 | South Africa | Black-footed cat | Africa 3 |
| FJ392380 | 2006 | South Africa | Yellow mongoose | Africa 3 |
| FJ392381 | 2006 | South Africa | Dog | Africa 3 |
| FJ392388 | 1995 | South Africa | Yellow mongoose | Africa 3 |
| FJ392379 | 1996 | South Africa | Yellow mongoose | Africa 3 |
| FJ392367 | 2001 | South Africa | Feline | Africa 3 |
| FJ392368 | 2001 | South Africa | Suricate | Africa 3 |
| FJ392369 | 2000 | South Africa | Slender mongoose | Africa 3 |
| FJ392370 | 2000 | South Africa | Bovine | Africa 3 |
| FJ392371 | 2002 | South Africa | Yellow mongoose | Africa 3 |
| FJ392373 | 2003 | South Africa | Slender mongoose | Africa 3 |
| FJ392385 | 1990 | South Africa | Yellow mongoose | Africa 3 |
| JX088729 | 2007 | South Africa | Bovine | Africa 3 |
| JX088730 | 2007 | South Africa | Bovine | Africa 3 |
| JX088731 | 2007 | South Africa | Slender mongoose | Africa 3 |
| JX088732 | 2006 | South Africa | Wildcat | Africa 3 |
| JX987743 | 2011 | Nepal | Dog | Arctic-related |
| GU371902 | 2009 | India | Dog | Arctic-related |
| AY352462 | 1998 | Russia | Human | Arctic-related |
| AY352486 | 1995 | Russia | Wolf | Arctic-related |
| AY730597 | 2004 | South Korea | Dog | Arctic-related |
| EU086198 | 1998 | Nepal | Mongoose | Arctic-related |
| U22654 | 1981 | Greenland | Arctic fox | Arctic-related |
| EU086209 | 2001 | Vietnam | Dog | Asian |
| EU086194 | 2002 | Laos | Dog | Asian |
| GQ303555 | 2009 | Thailand | Human | Asian |
| JQ040599 | 2010 | China | Dog | Asian |
| JX276406 | 2011 | China | Dog | Asian |
| EU159363 | 2004 | China | Dog | Asian |
| FJ719751 | 2008 | China | Ferret badger | Asian |
| EU086166 | 1999 | Myanmar | Dog | Asian |
| EU086168 | 1998 | Cambodia | Dog | Asian |
| EU086172 | 1998 | Cambodia | Dog | Asian |
| EU086173 | 1998 | China | Dog | Asian |
| EU086186 | 2005 | China | Dog | Asian |
| EU086192 | 2003 | Indonesia | Dog | Asian |
| EU086205 | 2004 | Philippines | Human | Asian |
| EU086208 | 1983 | Thailand | Human | Asian |
| EU086210 | 2001 | Viet Nam | Dog | Asian |
| FJ228493 | 2003 | Mexico | Vampire bat | Bat |
| KC758860 | 1996 | Argentina | Vampire bat | Bat |
| FJ228492 | 2002 | El Salvador | Human | Bat |
| JF693469 | 2002 | Colombia | Vampire bat | Bat |
| JF693475 | 1995 | Colombia | Vampire bat | Bat |
| EF363733 | 2005 | Brazil | Human | Bat |
| EU886635 | 2005 | Germany | Red fox | Cosmopolitan |
| EU038107 | 2005 | Nigeria | Dog | Cosmopolitan |
| FJ561731 | 2008 | China | Dog | Cosmopolitan |
| KJ744305 | 2011 | South Africa | Dog | Cosmopolitan |
| KJ744310 | 2011 | South Africa | Domestic dog | Cosmopolitan |
| KJ744309 | 2010 | South Africa | Goat | Cosmopolitan |
| JF747613 | 2008 | South Africa | Domestic dog | Cosmopolitan |
| JF747614 | 2008 | South Africa | Domestic dog | Cosmopolitan |
| HM179504 | 2006 | South Africa | Domestic dog | Cosmopolitan |
| HM179507 | 2005 | South Africa | Black-backed jackal | Cosmopolitan |
| HM179508 | 2005 | South Africa | Bat-eared fox | Cosmopolitan |
| KT336432 | 2012 | South Africa | Dog | Cosmopolitan |
| KT336436 | 2012 | South Africa | Dog | Cosmopolitan |
| KT336437 | 2012 | South Africa | Dog | Cosmopolitan |
| AF033905 | 1996 | France | Sheep | Cosmopolitan |
| U22841 | 1991 | Mexico | Human | Cosmopolitan |
| U42702 | 1991 | Germany | Red fox | Cosmopolitan |
| U42705 | 1981 | Serbia | Cattle | Cosmopolitan |
| U42706 | 1986 | Bosnia | Red fox | Cosmopolitan |
| U43025 | 1991 | Hungary | Human | Cosmopolitan |
| U43432 | 1991 | Estonia | Raccoon dog | Cosmopolitan |
| HM179505 | 2004 | South Africa | Domestic dog | Cosmopolitan |
| AB517659 | 2003 | Brazil | Domestic dog | Cosmopolitan |
| AB517660 | 2001 | Brazil | Fox | Cosmopolitan |
| HQ450386 | NA | Mexico | Dog | Cosmopolitan |
| U22483 | 1987 | Iran | Wolf | Cosmopolitan |
| U22484 | 1986 | Mozambique | Dog | Cosmopolitan |
| U22627 | 1979 | Egypt | Human | Cosmopolitan |
| U22629 | 1986 | Gabon | Dog | Cosmopolitan |
| U22839 | 1972 | Yugoslavia | Fox | Cosmopolitan |
| U22638 | 1979 | Zaire | Dog | Cosmopolitan |
| U22642 | 1990 | Morocco | Human | Cosmopolitan |
| U22643 | 1982 | Algeria | Dog | Cosmopolitan |
| U22645 | 1992 | Tanzania | Dog | Cosmopolitan |
| U22649 | 1992 | Namibia | Jackal | Cosmopolitan |
| U22650 | 1992 | Central African Republic | Dog | Cosmopolitan |
| JX944569 | 2009 | Nepal | Cattle | Indian subcontinent |
| JX944597 | 2009 | Nepal | Dog | Indian subcontinent |
| AY138550 | 2001 | Sri Lanka | Cattle | Indian subcontinent |
| AY138550 | 2001 | Sri Lanka | Bovine | Indian subcontinent |
