# Supplementary material for: Reverse transcription recombinase polymerase amplification assay for rapid detection of canine associated rabies virus in Africa
Source: PLoS One. 2019 Jul 5;14(7):e0219292. doi: 10.1371/journal.pone.0219292 (PMC6611627; doi:10.1371/journal.pone.0219292)
Supplement: S2 Table — (DOCX) [file pone.0219292.s002.docx]

**S2 Table. Evaluation of the mismatches between the RT-RPA primer and probe set binding regions to rabies-related lyssaviruses**

| Virus^a^ | Forward primer | | | | Reverse primer | | | | Probe | | | | Combined^b^ | RPA result | Time delay^c^ |
| --- | --- | --- | --- | --- | --- | --- | --- | --- | --- | --- | --- | --- | --- | --- | --- |
|  | 5’ | Internal | 3’ | Total % | 5’ | Internal | 3’ | Total % | 5’ | Internal | 3’ | Total % | Total % |  |  |
| ARAV | 2 | 3 | 1 | 19 | 4 | 1 | 1 | 20 | 1 | 2 | 1 | 8 | 15 | Pos | 5 |
| DUVV | 3 | 2 | 3 | 26 | 4 | 1 | 1 | 20 | 1 | 2 | 2 | 10 | 18 | Neg | N/A |
| EBLV-1 | 1 | 3 | 2 | 19 | 2 | 0 | 2 | 13 | 2 | 1 | 2 | 10 | 14 | Neg | N/A |
| EBLV-2 | 3 | 4 | 1 | 25 | 3 | 1 | 1 | 17 | 1 | 1 | 3 | 10 | 17 | Pos | 15 |
| IKOV | 2 | 3 | 3 | 25 | 4 | 2 | 1 | 23 | 1 | 1 | 3 | 10 | 19 | Neg | N/A |
| IRKV | 2 | 2 | 2 | 19 | 3 | 1 | 2 | 20 | 2 | 1 | 1 | 8 | 15 | Pos | 13 |
| KHUV | 4 | 3 | 0 | 23 | 4 | 2 | 2 | 27 | 1 | 1 | 3 | 10 | 19 | Pos | 15 |
| LBV A | 0 | 2 | 2 | 13 | 2 | 0 | 3 | 17 | 0 | 2 | 1 | 6 | 11 | Pos | 12 |
| LBV C | 1 | 1 | 3 | 16 | 3 | 1 | 0 | 13 | 1 | 2 | 3 | 13 | 14 | Pos | 16 |
| LBV D | 1 | 2 | 1 | 13 | 2 | 1 | 1 | 13 | 2 | 3 | 2 | 15 | 14 | Pos | 12 |
| MOKV | 1 | 3 | 2 | 19 | 2 | 2 | 2 | 20 | 2 | 1 | 1 | 8 | 15 | Pos | 2 |
| SHIBV | 2 | 3 | 3 | 26 | 4 | 1 | 1 | 20 | 1 | 0 | 2 | 6 | 16 | Neg | N/A |
| WCBV | 1 | 4 | 1 | 19 | 1 | 1 | 3 | 17 | 0 | 0 | 1 | 2 | 11 | Pos | 12 |

^a^ Genbank accession numbers can be found in Fig 5

^b^ Total percentage of mismatches across primer and probe binding sites (n=108)

^c^ Estimated additional time required for a positive RT-RPA result (based on qRT-PCR quantification)
